# Prevalence and Determinants of Undernutrition Among Children Under Five in Coastal Bangladesh: A Community‐Based Study

**DOI:** 10.1002/fsn3.71573

**Published:** 2026-02-20

**Authors:** Shahinur Akter, Aranya Siriphon, Arratee Ayuttacorn, Waraporn Boonchieng

**Affiliations:** ^1^ Faculty of Social Sciences Chiang Mai University Chiang Mai Thailand; ^2^ Sociology Discipline, Social Science School Khulna University Khulna Bangladesh; ^3^ Department of Sociology and Anthropology, Faculty of Social Sciences Chiang Mai University Chiang Mai Thailand; ^4^ Department of Social Sciences and Development Chiang Mai University Chiang Mai Thailand; ^5^ Faculty of Public Health Chiang Mai University Chiang Mai Thailand

**Keywords:** child undernutrition, coastal Bangladesh, determinants, prevalence, stunting, underweight, wasting

## Abstract

This study investigates the prevalence and determinants of undernutrition among children under five in coastal Bangladesh employing the Social Ecological Model (SEM). A cross‐sectional survey was conducted among 348 randomly selected caregivers from six villages in *Dacope* upazila of Khulna district, between July and October 2024. Undernutrition in children was assessed using World Health Organization (WHO) standards for stunting, wasting, and underweight. Findings revealed high levels of undernutrition prevalence among children under five, with 56.3% severe stunting and 33.3% moderate stunting, 16.4% severe wasting and 40.5% moderate wasting, and 28.7% severe underweight and 59.2% moderate underweight, respectively. Multivariate analyses demonstrated that stunting was significantly associated with child age, birth weight, caregivers' occupation and mass media exposure, education of the household head, household food insecurity, vulnerability, and membership of non‐governmental organization (NGO). Wasting prevalence was influenced by child age and sex, exclusive breastfeeding, feeding practices, caregivers' education, occupation, income, religion, and mass media exposure, household vulnerability, NGO membership, and place of residence. Underweight prevalence was primarily associated with child age, sex, birth weight, caregivers' income, and exposure to natural disasters. This study emphasizes the need for integrated, multi‐level strategies to address child undernutrition. Local actions should prioritize young and low‐birth‐weight children through improved feeding practices, maternal education, and livelihood support in disaster‐prone areas, while national policies must embed nutrition within health, poverty alleviation, and social protection programs. Globally, climate‐resilient and context‐specific nutrition policies, supported by WHO, United Nations Children's Fund (UNICEF), and World Food Programme (WFP), are vital to ensuring sustainable and equitable child health outcomes.

## Graphical abstract

This study examined undernutrition among children under five in coastal Bangladesh employing the Social Ecological Model. A cross‐sectional survey of 348 caregivers from six villages in *Dacope* upazila (July–October 2024) revealed high levels of stunting, wasting, and underweight prevalence. Multi‐level determinants of childhood undernutrition were identified across personal, interpersonal, community, and policy levels. The study recommends integrated strategies spanning local, national, and global policies, along with cross‐country collaborations, to promote equitable and sustainable improvements in child nutrition and health outcomes.

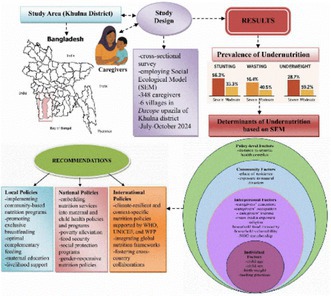

## 1 Introduction

Childhood undernutrition remains a significant public health issue and a growing policy concern worldwide, especially in low‐ and middle‐income countries (LMICs). It leads to impaired physical and mental development and is a substantial contributor to child morbidity and mortality (Rahman et al. 2020). While undernutrition is a complex issue, stunting (low height‐for‐age), wasting (low weight‐for‐height), and underweight (low weight‐for‐age) are internationally accepted indicators used to assess child undernutrition (World Health Organization 2023). The World Health Organization (WHO) defines stunting, wasting, and underweight as *Z*‐scores below −2 standard deviations from the median of the WHO Child Growth Standards (De Onis et al. 2019). In 2022, approximately 4.9 million children under five died globally (World Health Organization 2025), and nearly half of these deaths were attributed to stunting, wasting, and underweight, with the majority occurring in LMICs (World Health Organization 2024).

In 2022, 149 million children under five (22.3%) were stunted, while 45 million (6.8%) suffered from wasting, and 390 million were underweight worldwide (World Health Organization 2024). Over the past 20 years, undernutrition has become a significant child health concern in LMICs due to its high correlation with child mortality (Das et al. 2020). LMICs bear a large portion of this burden (UNICEF 2023), especially in South Asia (SA), where stunting and wasting are extremely common, affecting 31.4% and 14.8% of children under five, respectively (World Health Organization 2023). Like other South Asian countries, child undernutrition is still a significant public health issue in Bangladesh and a leading cause of death for children under five (Chowdhury et al. 2020). Growth failure is currently quite common in children under five in Bangladesh, with 40% of children affected by one or more types of stunting, wasting or underweight and accounting for over 50% of child fatalities (Chowdhury et al. 2021). According to the most recent Bangladesh Demographic and Health Survey (BDHS) 2022, the nationwide prevalence of stunting is 24%, wasting (11%), and underweight (22%) (National Institute of Population Research and Training (NIPORT) and ICF 2023).

Childhood undernutrition is a multifaceted issue that is significantly affected by different factors at individual, household, and community levels (Das and Gulshan 2017). Among individual factors, child age (Jama 2025; Kassie and Workie 2020), sex (Adedokun and Yaya 2021; Elmighrabi et al. 2024), birth weight (Katoch 2022; Oswal et al. 2025; Yigezu et al. 2024), birth order (Jama 2025; Tamanna et al. 2025; Tesema et al. 2021), birth type (Burki 2025; Kundu et al. 2024; Tesema et al. 2021), and place of delivery (Tamanna et al. 2025) were evident as significant determinants of childhood undernutrition. Additionally, several parental factors have been identified as significant determinants of child undernutrition, such as parental education (Khanam et al. 2019; Mohammed and Asfaw 2018; Tesema et al. 2021), parental occupation (Murarkar et al. 2020; Tamanna et al. 2025), mother's body mass index (BMI) (Elmighrabi et al. 2024; Yigezu et al. 2024), maternal media exposure (Oswal et al. 2025; Tamanna et al. 2025), mother's autonomy (Paul and Saha 2022), and religion (Banerjee and Shirisha 2023; Kundu et al. 2024).

Household‐level determinants of child undernutrition include factors like family income (Murarkar et al. 2020), household wealth (Kundu et al. 2024; Tamanna et al. 2025), family structure (Murarkar et al. 2020), family size (Kundu et al. 2024; Yigezu et al. 2024), and household food insecurity (Bogin 2022; Katoch 2024). At the community‐level, regional disparities (Kassie and Workie 2020; Khanam et al. 2019), rural–urban differences (Khanam et al. 2019; Kundu et al. 2024), inadequate healthcare services (Bogin 2022), and inadequate sanitation and hygiene practices (Jama 2025; Vijay and Patel 2024) have been identified as significant contributors. In addition, child feeding practices such as exclusive breastfeeding (Jama 2025; Murarkar et al. 2020; Yigezu et al. 2024), delayed initiation of complementary feeding (Vijay and Patel 2024), insufficient dietary intake (Bogin 2022; Vijay and Patel 2024), and feeding frequency (Bogin 2022; Yigezu et al. 2024) play a critical role. Policy level factors including long distance to health facilities (Shahid et al. 2022) further exacerbate child undernutrition. Considering the multidimensional nature of these determinants, addressing childhood undernutrition, particularly stunting, wasting, and underweight in LMICs necessitates a comprehensive strategy that not only targets these recognized factors but also investigates the less explored dimensions of this complex issue.

Undernutrition in children can have serious repercussions beginning with a weakened immune system that increases the risk of communicable diseases, premature death, and impaired physical and cognitive development, and the continuation of the intergeneration cycle of malnutrition (John‐Joy Owolade et al. 2022; World Health Organization 2023). Thus addressing undernutrition is paramount to ensuring healthy growth and development in children (Vijay and Patel 2024) and identifying the potential factors contributing to childhood undernutrition is a vital initial step toward accelerating the reduction rate. In response to this persistent issue, Sustainable Development Goal (SDG) 2.2 has established a target of eradicating stunting and wasting in children under five by the year 2030 (UNICEF 2023; World Health Organization 2023). Although several national‐level studies conducted in Bangladesh have predominantly relied on secondary data (Chowdhury, Rahman, et al. 2022; Chowdhury, Chakrabarty, et al. 2022; Khan et al. 2024; Khanam et al. 2019; Tamanna et al. 2025), limited attention has been given to community‐level prevalence and context‐specific determinants of childhood undernutrition (Akter and Nishu 2025; Jubayer et al. 2022). This gap in localized evidence hinders the implementation of effective interventions. Therefore, a comprehensive understanding of community‐based dynamics is essential for designing targeted strategies to reduce stunting, wasting, and underweight among children under five and to improve child health outcomes. In this context, the present study aims to investigate the prevalence and determinants of undernutrition in the form of stunting, wasting, and underweight among children under five in the southwestern coastal region of Bangladesh.

## 2 Theoretical Framework

Urie Bronfenbrenner first introduced the Social Ecological Model (SEM) in 1970s as a conceptual framework for understanding human development, and it was later formalized as a theory in 1980s (Bronfenbrenner 2000). The SEM offers a holistic lens for examining childhood undernutrition by recognizing that health outcomes are shaped by multiple, interacting levels of influence such as individual, interpersonal, community, organizational, and policy (McLeroy et al. 1988). Its versatility has led to its application in addressing complex public health challenges such as suicide, violence, childhood communicable diseases and obesity, vaccination uptake, and cancer screening (Akter et al. 2025; Golden and Earp 2012). The model emphasizes the dynamic interplay between individuals and their environments, illustrating how behaviors and health outcomes are influenced by individual level attributes (e.g., age, sex, education, knowledge, attitudes, and skills); interpersonal factors (e.g., family, peers, and social support); community‐level contexts (e.g., local norms, institutions, and environmental settings); and policy‐level determinants (e.g., institutional or governmental decisions shaping health access and equity).

**FIGURE 1:**
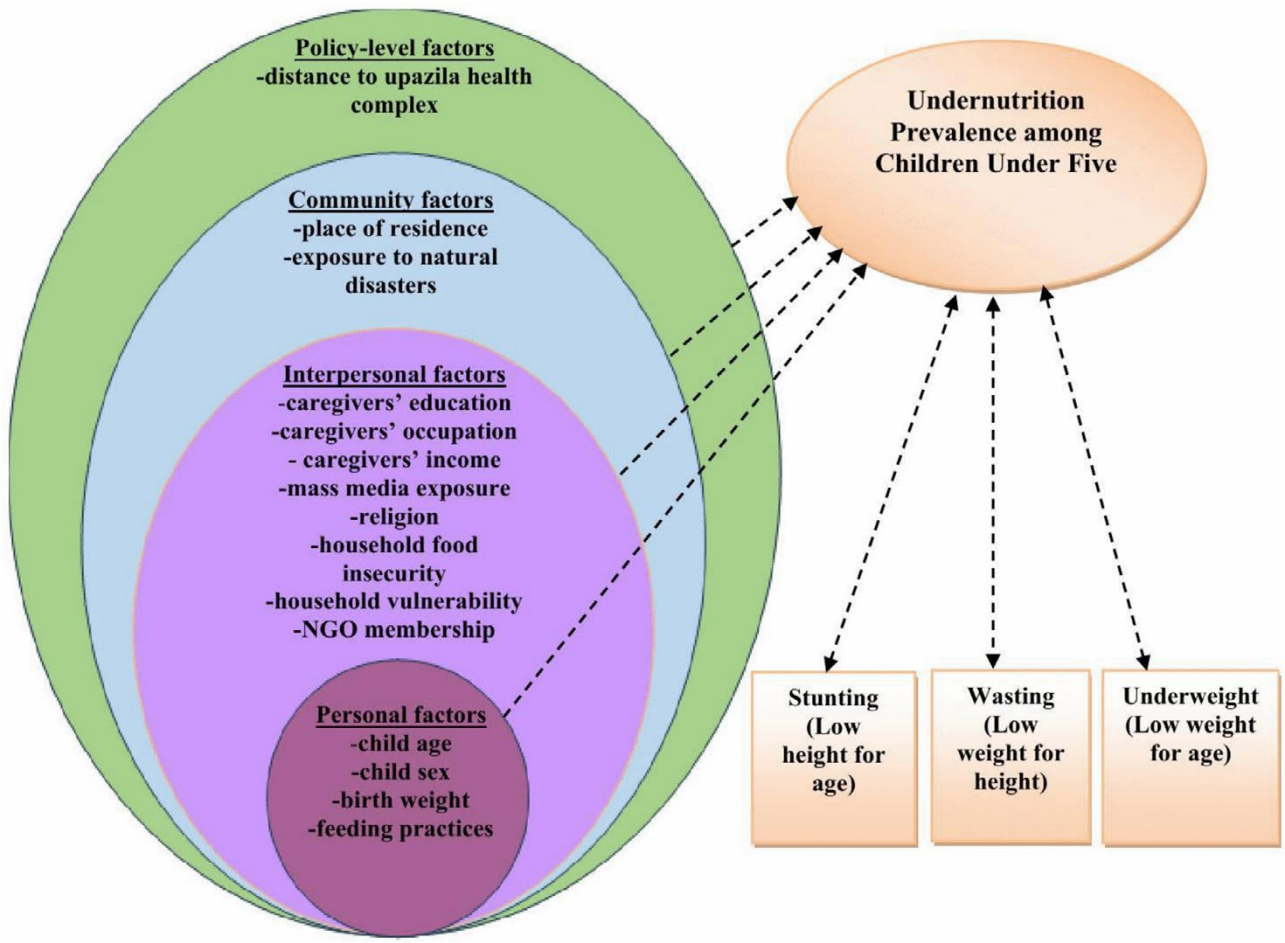
Determinants of undernutrition among children under five based on the Social Ecological Model.

Guided by the SEM, this study examined determinants of childhood undernutrition in the form of stunting, wasting, and underweight among children under five in coastal Bangladesh. Factors were categorized into four levels: individual (child age, sex, birth weight, exclusive breastfeeding practices, and feeding frequency); interpersonal, comprising caregiver and household characteristics (caregivers' education, occupation, income, and mass media exposure, religion, education of the household head, household food insecurity, vulnerability and NGO membership); community‐level variables (place of residence and exposure to natural disasters over the past six years); and policy‐level determinants (distance to upazila health complex) (see Figure 1). This structured approach enabled a comprehensive understanding of the multifactorial nature of child undernutrition within its ecological context.

## 3 Methods

### 3.1 Study Area

**Map 1:**
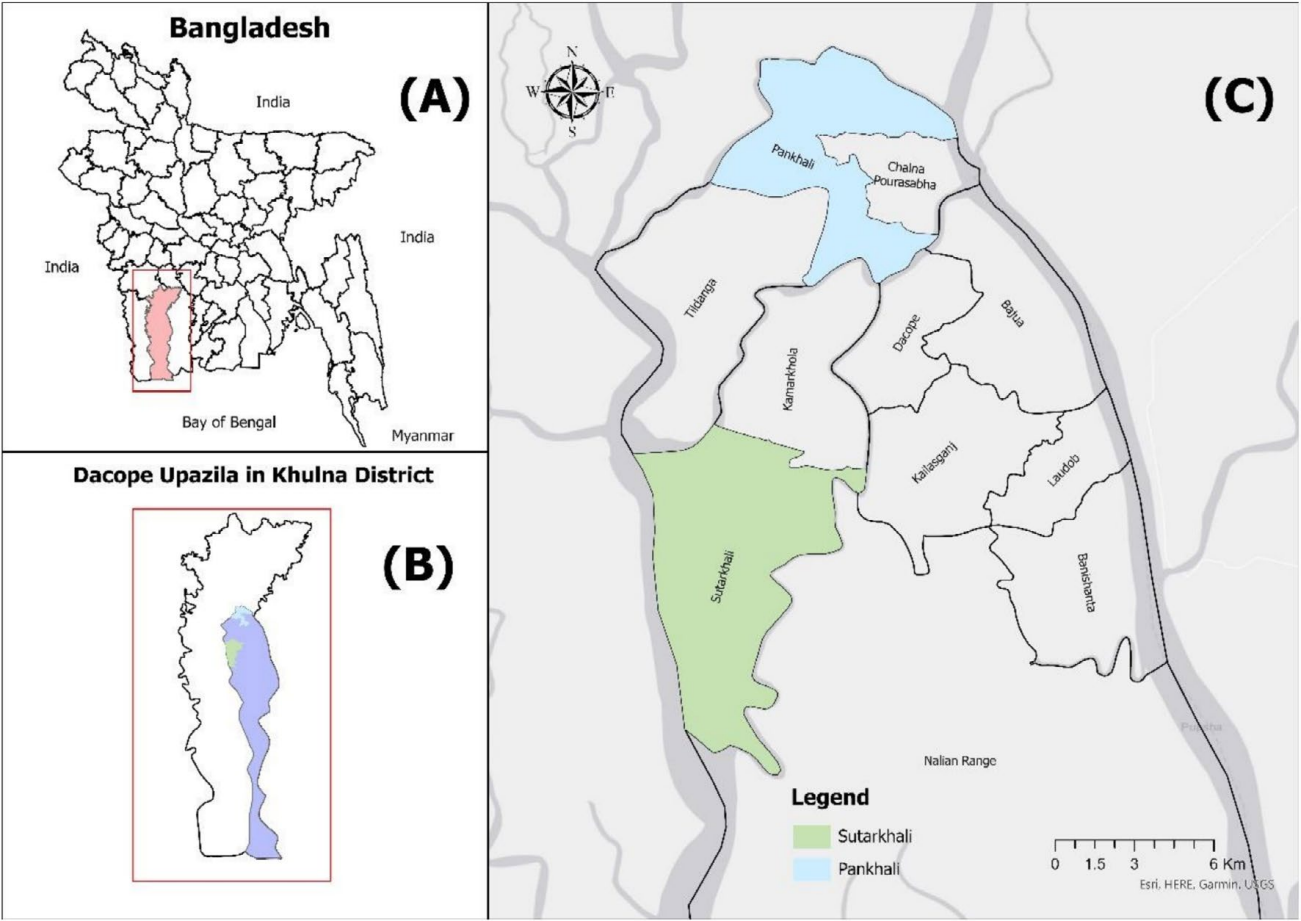
Map of the study area.

This explanatory study was conducted in *Dacope* upazila (sub‐district) of Khulna district in Bangladesh, utilizing a cross‐sectional survey design. *Dacope* upazila has been chosen for this study (see Map 1) due to its proximity to the Bay of Bengal and high exposure to recurrent coastal hazards. This upazila spans 991.6 km^2^ with a population of 1,59,369 (density 161/km^2^) and 42,186 households, averaging 3.76 members per household (Bangladesh Bureau of Statistics 2025). *Dacope* is characterized by rural settlements (71.45%), nature‐dependent livelihoods, and widespread housing and infrastructure deficits (77% of residents live in *Katcha* houses), whereas drinking water sources remain precarious as 34.11% rely on rainwater, 31.55% on surface water, and 25.95% on tube wells (Bangladesh Bureau of Statistics 2025). Sanitation facilities are similarly limited, with only 35.4% using improved facilities and 37.11% using pit latrines/pit latrines with slabs (Bangladesh Bureau of Statistics 2025). To capture the scenario of child undernutrition in southwestern coastal Bangladesh, two unions, namely *Pankhali* and *Sutarkhali*, were purposively selected and six villages (*Pankhali*, *Hoglabunia*, *Katabunia, Sutarkhali*, *Nolian*, and *Kalabogi* villages), 3 from each union, were chosen as study sites. This setting reflects the socioeconomic marginalization and environmental risk characteristics of Bangladesh's southwestern coastal zone.

### 3.2 Participants

In this research, participants were chosen based on the following inclusion criteria: (i) participants were caregivers, including mothers, fathers, or grandparents responsible for the care of the children; (ii) the children's age ranged from 6 months to 59 months; (iii) caregivers were required to live with the children in the study area for a minimum of five consecutive years. The residency requirement ensures stable living conditions, fosters their ties within the community, and ensures a comprehensive understanding of the complex nature of childhood undernutrition and the various factors that affect its prevalence in coastal Bangladesh.

### 3.3 Sampling and Sample Size Determination

A household census was conducted to collect information about the population in the study area, strictly adhering to the inclusion criteria for the participants. Following the census, a population list was created, and each participant was assigned a serial number. In this research, a total of 3650 caregivers were included based on the census conducted. Using Cochran's random sampling formula (Cochran 1977), a sample size of 348 caregivers was calculated with 95% confidence level and 5% margin of error. The required sample size was initially calculated using the standard formula for a single population proportion: $n_{0}=\frac{Z^{2}\mathrm{pq}}{e^{2}}$, where Z denotes the Z‐value corresponding to a 95% confidence level (1.96), *p* represents the estimated proportion of the attribute present in the population (0.5), *q* = 1—*p*, and *e* indicates the margin of error (0.05). Substituting these values yielded an initial sample size of 384. Given that the total population (N) was 3650, the sample size was adjusted using the finite population correction formula: $n=\frac{n_{0}}{1+\left(\frac{n_{0}-1}{N}\right)}$, resulting in a final sample size of 348 participants. Subsequently, we selected 58 participants from each village (with each village is treated as a stratum) utilizing a disproportionate stratified random sampling technique for equal representation across the six selected villages in the study area. To promote fairness and ensure randomization, participants were randomly chosen from the population list.

### 3.4 Data Collection

Data were collected using a semi‐structured interview schedule comprising several sections that captured information on sociodemographic characteristics of caregivers and children, household details, vulnerability context, asset ownership, household food insecurity, and the prevalence of child undernutrition, including stunting, wasting, and underweight among children under five. The quality of the data collection tool was evaluated by three experts who reviewed its content and assessed the index of item‐objective congruence (IOC) values. Based on their feedback, necessary revisions were incorporated to enhance clarity and relevance. Field data collection was conducted by the first author with support from trained data collectors through face‐to‐face interviews from July to October 2024, following institutional ethical approval. Written informed consent was obtained from all the participants, who were assured of the confidentiality and anonymity of their responses.

Data collection was carried out at the household level following a replacement sampling approach to enhance the accuracy and reliability of the findings. For households with more than one child under the age of five, information was collected specifically for the youngest child to obtain a more precise understanding of child undernutrition and its determinants. To accurately assess the prevalence of stunting, wasting and underweight, standardized anthropometric measurement tools were employed, specifically vertical height scales and digital weighing devices. Trained data collectors measured the height of each child by ensuring proper alignment of the head, shoulders, buttocks, and heels against a flat surface. Height was recorded in centimeters and weight in kilograms to ensure accuracy and facilitate comprehensive analysis.

## 4 Measures

### 4.1 Outcome Variable

Undernutrition among children under five served as the outcome variable in this study. It was assessed using three internationally recognized anthropometric indicators of physical growth such as stunting, wasting, and underweight as defined by WHO Child Growth Standards (De Onis et al. 2019). Z‐scores were manually computed based on the WHO reference growth charts for boys and girls aged 0–5 years to classify stunting, wasting, and underweight. Stunting and wasting were categorized into three categories such as severely stunted and wasted (Z‐score is below −3.0), moderately stunted and wasted (Z‐score is below −2.0), and normal height and weight (Z‐score is “0” to less than +2). Besides, underweight was categorized into four categories such as severely underweight (Z‐score is below −3.0), moderately underweight (Z‐score is below −2.0), normal weight (Z‐score is “0” to less than +2), and overweight (Z‐score is above +2.0) (World Health Organization and UNICEF 2009).

### 4.2 Explanatory Variables

The explanatory variables in this study were organized according to the SEM into individual, interpersonal, community, and policy‐level factors.

#### 4.2.1 Individual Factors

Individual‐level factors included child‐specific characteristics such as age (categorized as 6–12, 13–24, 25–36, 37–48, and 49–59 months), sex (girl/boy), birth order (1, 2, and ≥ 3), and type of delivery (cesarean or normal). Birth weight was classified following WHO guidelines into underweight (< 2.5 kg), normal weight (2.5–3.9 kg), and overweight (≥ 4 kg) (World Health Organization 2004). Feeding practices were assessed based on feeding frequency, categorized as ≤ 4 times, 5–6 times, and ≥ 7 times per day, and whether the child was exclusively breastfed during the first 6 months of life (no and yes).

#### 4.2.2 Interpersonal Factors

Interpersonal factors encompassed caregiver and household characteristics. Caregiver‐related variables included educational attainment (non‐literate, primary [1–5 years], secondary [6–10 years], and higher education [≥ 11 years]), occupation (farming/fishing/fish cultivation, day labor, housewife, bamboo crafting, and others such as business, job, tailoring), monthly income in Bangladeshi Taka [BDT] (no income, < 5000, and ≥ 5000), and religion (Muslim and non‐Muslim). Exposure to mass media was coded as yes = 1 (if caregivers had access to any form of media, including mobile phones, radio, television, magazines or newspapers) and no = 0 (if caregivers lacked access to these sources).

Household‐level information included the education of the household head (non‐literate, primary [1–5 years], secondary [6–10 years], and higher education [≥ 11 years]), and NGO membership (no/yes). A household asset index was used comprising 27 items from the BDHS (National Institute of Population Research and Training 2020), with responses coded dichotomously (yes = 1 and no = 0). Total asset scores ranged from 0 to 16, with a mean of 6, and were classified as low (< 6), moderate (6–10), and high (11–16). Household food insecurity was measured using the Household Food Insecurity Access Scale (HFIAS) developed by Coates et al. (2007) and categorized as food secure, mildly food insecure, moderately food insecure, or severely food insecure. Household vulnerability was assessed using an adapted version of the Hahn et al. (2009) vulnerability index, incorporating seven components: sociodemographic profile, livelihood strategies, social networks, health, food, water, and disasters. The index consisted of 23 indicators scored from 0 to 1, yielding a total score between 2 and 17 (mean = 10). Based on these scores, household vulnerability was categorized into low (< 10), moderate (10–13), and high (14–17).

#### 4.2.3 Community‐Level Factors

Community‐level variables included place of residence, categorized into six villages (*Pankhali*, *Hoglabunia*, *Katabunia*, *Sutarkhali*, *Nolian*, and *Kalabogi*). Additionally, the frequency of natural disasters experienced in the locality over the past 6 years was classified as ≤ 6 events or ≥ 7 events.

#### 4.2.4 Policy‐Level Factors

Policy‐level factors were represented by the distance from the household to the nearest Upazila Health Complex (UHC). This distance was measured in kilometers (km) and grouped into three categories: < 10, 10–20, and > 20 km.

### 4.3 Data Analysis

Following the field survey, data were analyzed using the Statistical Package for the Social Sciences (SPSS) version 21. Descriptive statistics, including percentage analyses, were performed to assess the prevalence of undernutrition specifically stunting, wasting, and underweight among children under five. To identify the key determinants of these forms of undernutrition, multivariate logistic regression analyses were conducted. The outcome of regression analyses was reported as adjusted odds ratio (AOR) with 95% confidence interval (CI) and a significance level of *p* < 0.10.

## 5 Results

### 5.1 Prevalence of Stunting, Wasting, and Underweight Among Children Under Five

Table 1 presents the prevalence of stunting, wasting, and underweight among children under five in coastal Bangladesh. Findings indicated that stunting was highly prevalent, with 56.3% of children classified as severely stunted, 33.3% as moderately stunted, and only 10.4% exhibiting normal height‐for‐age. In terms of wasting, the majority of children were within the normal weight (43.1%), while 40.5% were moderately wasted and 16.4% were severely wasted. Regarding underweight, around 60% of children were moderately underweight, 28.7% were severely underweight, 9.8% had normal weight, and 2.8% were overweight.

**TABLE 1:** Prevalence of stunting, wasting, and underweight among children under five.

Child undernutrition	Frequency	Percent (%)
Stunting status		
Normal height	36	10.4
Moderately stunted	116	33.3
Severely stunted	196	56.3
Wasting status		
Normal weight	150	43.1
Moderately wasted	141	40.5
Severely wasted	57	16.4
Underweight status		
Normal weight	34	9.8
Overweight	8	2.3
Moderately underweight	206	59.2
Severely underweight	100	28.7

### 5.2 Determinants of Stunting Among Children Under Five

Multinominal logistic regression analysis was conducted to identify the determinants of stunting among children under five (see Table 2). Stunting served as the outcome variable and was categorized as normal height (reference category), moderately stunted, and severely stunted. Explanatory variables encompassed personal (child), interpersonal (caregivers and household), community and policy‐level factors. The analysis indicated that individual factors such as child age and birth weight, and interpersonal factors including caregivers' occupation, mass media exposure, education of the household head, household food insecurity and vulnerability, and NGO membership were significantly associated with stunting among children under five. However, community and policy‐level factors were not significantly related to stunting prevalence.

**TABLE 2:** Multivariate logistic regression analysis of stunting and its determinants.

Variables		Moderately stunted			Severely stunted		
		AOR	95% CI (Lower‐upper)	*p*	AOR	95% CI (Lower‐upper)	*p*
**Individual factors**							
Age of the children							
6–12 months		0.092	0.014–0.620	0.014**	0.451	0.073–2.791	0.392
13–24 months		0.358	0.054–2.363	0.286	1.934	0.308–12.143	0.482
25–36 months		0.384	0.085–1.745	0.215	0.687	0.152–3.115	0.627
37–48 months		0.585	0.126–2.716	0.493	0.870	0.188–4.015	0.858
49–59 months ^(R)^		1.00			1.00		
Sex of the children							
Girl		0.717	0.261–1.972	0.519	0.977	0.368–2.596	0.963
Boy^(R)^		1.00			1.00		
Birth order							
1		0.383	0.034–4.334	0.438	0.292	0.026–3.259	0.317
2		0.724	0.064–8.149	0.794	0.600	0.054–6.661	0.677
≥ 3^(R)^		1.00			1.00		
Birth weight							
Underweight		24.871	1.259–491.376	0.035**	48.222	2.652–876.814	0.009***
Normal weight		5.660	0.463–69.219	0.175	9.556	0.826–110.498	0.071*
Overweight^(R)^		1.00			1.00		
Type of delivery							
Caesarian		1.244	0.436–3.545	0.683	1.189	0.432–3.273	0.738
Normal^(R)^		1.00			1.00		
Excusive breastfeeding in early 6 months							
No		0.857	0.208–3.537	0.831	1.165	0.296–4.580	0.827
Yes^(R)^		1.00			1.00		
Feeding frequency							
≤ 4 times		0.380	0.057–2.527	0.317	0.600	0.097–3.715	0.582
5–6 times		0.993	0.135–7.329	0.995	0.973	0.140–6.746	0.978
≥ 7 times^(R)^		1.00			1.00		
**Interpersonal factors**							
Caregivers' education							
	Non literate	4,899,107.464	0.000	0.985	335,705.492	0.000	0.988
	Primary	1.715	0.290–10.143	0.552	0.978	0.182–5.252	0.979
	Secondary	1.589	0.343–7.363	0.554	0.777	0.187–3.231	0.729
	Higher education^(R)^	1.00			1.00		
**Caregivers' occupation**							
	Farming/fishing/fish cultivation	2.226	0.077–64.048	0.641	0.272	0.010–7.520	0.442
	Day labor	310,683.613	0.000	0.990	2,949,691.109	0.000	0.988
	Housewife	15,313,228.455	0.000	0.983	3,014,104,438,094.203	4,508,957,608,419.863–201,483,942,920,957.000	< 0.001***
	Bamboo crafting	0.715	0.021–24.728	0.853	0.673	0.021–21.906	0.824
	Others (Business/job/tailoring)^(R)^	1.00			1.00		
Caregivers' monthly income (in BDT)							
	No income	1.082E‐007	0.000	0.984	1.752E‐013	1.752E‐013	—
	< 5000	1.770	0.118–26.573	0.680	2.693	0.186–38.955	0.467
	≥ 5000^(R)^	1.00			1.00		
Religion							
	Non‐Muslim	0.966	0.207–4.511	0.965	2.328	0.543–9.981	0.255
	Muslim^(R)^	1.00			1.00		
Caregivers' exposure to mass media							
	No	4.585	1.367–15.381	0.014**	3.101	0.937–10.258	0.064*
	Yes ^(R)^	1.00			1.00		
Education of the household head							
	Non literate	2,430,948.249	0.000	0.976	155,618.043	0.000	0.977
	Primary	2.959	0.591–14.806	0.187	4.951	1.033–23.725	0.045**
	Secondary	1.946	0.501–7.556	0.336	1.715	0.448–6.563	0.431
	Higher education^(R)^	1.00			1.00		
Household assets							
	Low	0.140	0.006–3.202	0.218	0.126	0.006–2.587	0.179
	Moderate	0.245	0.013–4.748	0.352	0.216	0.013–3.715	0.291
	High^(R)^	1.00			1.00		
**Interpersonal factors**							
Household food insecurity							
Food secure household		0.409	0.070–2.373	0.319	0.219	0.042–1.141	0.071*
Mildly food insecure household		0.388	0.064–2.350	0.303	0.296	0.054–1.614	0.160
Moderately food insecure household		1.304	0.251–6.784	0.752	1.045	0.215–5.084	0.957
Severely food insecure household^(R)^		1.00			1.00		
Household vulnerability							
Low		0.447	0.035–5.771	0.537	0.324	0.028–3.730	0.366
Moderate		0.223	0.019–2.564	0.228	0.116	0.011–1.196	0.070*
High^(R)^		1.00			1.00		
Household NGO membership							
No		3.207	1.030–9.700	0.039**	2.407	0.835–6.938	0.104
Yes^(R)^		1.00			1.00		
**Community‐level factors**							
Place of residence							
*Pankhali* village		1.900E‐013	0.000	0.984	1.115E‐013	0.000	0.982
*Hoglabunia* village		1.925E‐013	0.000	0.984	1.244E‐013	0.000	0.983
*Katabunia* village		2.661E‐013	0.000	0.984	1.662E‐013	0.000	0.983
*Sutarkhali* village		1.439E‐013	0.000	0.983	1.081E‐013	0.000	0.982
*Nolian* village		1.178E‐006	0.000	0.972	3.140E‐007	0.000	0.969
*Kalabogi* village^(R)^		1.00			1.00		
Frequency of natural disasters over the past 6 years							
≤ 6 events		1.949	0.445–8.531	0.376	2.448	0.576–10.399	0.225
≥ 7 events^(R)^		1.00			1.00		
**Policy‐level factors**							
Distance to upazila health complex							
< 10 km		9,094,239.106	0.000	0.991	58,176,196.447	0.000	0.990
10–20 km		2,711,446.224	0.000	0.992	19,348,882.076	0.000	0.991
> 20 km^(R)^		1.00			1.00		

Abbreviations: AOR = adjusted odds ratio; CI = confidence interval; R = reference category.

* Significant at 10% level.

** Significant at 5% level.

*** Significant at 1% level.

Children aged 6–12 months had 0.092 times lower odds of moderate stunting compared to children aged 49–59 months (AOR = 0.092; 95% CI: 0.014–0.620; *p* = 0.014). Birth weight was a significant determinant, with underweight children having markedly 24.871 times and 48.222 times higher odds of both moderate (AOR = 24.871; 95% CI: 1.259–491.376; *p* = 0.035) and severe stunting (AOR = 48.222; 95% CI: 2.652–876.814; *p* = 0.009) as well as children with normal birth weight having 9.556 times greater odds of severe stunting (AOR = 9.556; 95% CI: 0.826–110.498; *p* = 0.071) compared to those born overweight.

Besides, caregivers' occupation also found as a significant predictor as children of housewives had significantly greater odds of severe stunting (AOR = 3,014,104,438,094.203; 95% CI: 4,508,957,608,419.863–201,483,942,920,957.000; *p* < 0.001). Additionally, lack of exposure to mass media was associated with fourfold and threefold increase in the likelihood of moderate (AOR = 4.585; 95% CI: 1.367–15.381; *p* = 0.014) and severe stunting (AOR = 3.101; 95% CI: 0.937–10.258; *p* = 0.064). Children from households where the heads had only primary education had 4.51 times higher odds of severe stunting (AOR = 4.951; 95% CI: 1.033–23.725; *p* = 0.045) compared to household heads with higher education. Children from food‐secure households (AOR = 0.219; 95% CI: 0.042–1.141; *p* = 0.071) and moderate vulnerable households had lower odds of severe stunting (AOR = 0.116; 95% CI: 0.011–1.196; *p* = 0.070) than their counterparts. Moreover, absence of household NGO membership significantly increased the odds of moderate stunting (AOR = 3.207; 95% CI: 1.030–9.700; *p* = 0.039).

### 5.3 Determinants of Wasting Among Children Under Five

Multivariate logistic regression analysis was employed to determine the predictors of wasting among children under five (see Table 3). Wasting status was classified into normal weight (reference category), moderately wasted, and severely wasted. Explanatory variables included factors at the individual, interpersonal, community, and policy levels. The results indicated that individual factors such as child age, sex, exclusive breastfeeding in the early 6 months, and feeding frequency were significantly associated with wasting. Among interpersonal factors, caregivers' education, occupation, monthly income, religion, and exposure to mass media, household vulnerability, and NGO membership were significant predictors. Additionally, the community‐level factor, specifically place of residence, was also found to influence wasting prevalence among children under five.

**TABLE 3:** Multivariate logistic regression analysis of wasting and its determinants.

Variables	Moderately wasted			Severely wasted		
	AOR	95% CI (Lower‐upper)	*p*	AOR	95% CI (Lower‐upper)	*p*
**Individual factors**						
Age of the children						
6–12 months	0.143	0.047–0.433	0.001***	0.096	0.020–0.465	0.004***
13–24 months	0.176	0.067–0.466	< 0.001***	0.198	0.051–0.767	0.019**
25–36 months	0.388	0.160–0.939	0.036**	0.409	0.117–1.429	0.161
37–48 months	0.504	0.211–1.201	0.122	0.435	0.131–1.449	0.175
49–59 months^(R)^	1.00			1.00		
Sex of the children						
Girl	1.358	0.773–2.385	0.287	2.498	1.134–5.504	0.023**
Boy^(R)^	1.00			1.00		
Birth order						
1	0.847	0.336–2.136	0.725	1.431	0.388–5.274	0.590
2	1.233	0.504–3.021	0.646	0.480	0.125–1.843	0.285
≥ 3^(R)^	1.00			1.00		
Birth weight						
Underweight	4.021	0.691–23.383	0.121	4,603,804.141	0.000	0.986
Normal weight	3.587	0.708–18.174	0.123	2,182,384.077	0.000	0.987
Overweight^(R)^	1.00			1.00		
Type of delivery						
Caesarian	1.609	0.872–2.968	0.128	0.925	0.384–2.227	0.862
Normal^(R)^	1.00			1.00		
Excusive breastfeeding in early 6 months						
No	0.919	0.377–2.245	0.854	0.064	0.012–0.343	0.001***
Yes^(R)^	1.00			1.00		
Feeding frequency						
≤ 4 times	0.451	0.162–1.261	0.129	0.121	0.029–0.505	0.004***
5–6 times	0.637	0.225–1.806	0.396	0.126	0.031–0.521	0.004***
≥ 7 times^(R)^	1.00			1.00		
**Interpersonal factors**						
Caregivers' education						
Non literate	6.695	1.112–40.306	0.038**	0.953	0.056–16.130	0.973
Primary	1.536	0.513–4.597	0.443	0.258	0.059–1.132	0.073*
Secondary	1.738	0.640–4.723	0.278	0.497	0.133–1.855	0.298
Higher education^(R)^	1.00			1.00		
Caregivers' occupation						
Farming/fishing/fish cultivation	14.333	0.798–257.551	0.071	6.025	0.224–162.094	0.285
Day labor	1.100	0.116–10.433	0.934	6.352E‐007	0.000	0.988
Housewife	4.211E‐006	0.000	0.991	4.352E‐012	6.856E‐013–3.097E‐011	< 0.001***
Bamboo crafting	4.834	0.794–29.431	0.087*	0.304	0.010–9.060	0.491
Others (Business/job/tailoring) ^(R)^	1.00			1.00		
Caregivers' monthly income (in BDT)						
No income	263,791.157	0.000	0.991	548,986,218,739.585	548,986,218,739.585–548,986,218,739.585	—
< 5000	0.178	0.042–0.753	0.019**	0.274	0.024–3.128	0.297
≥ 5000^(R)^	1.00			1.00		
Religion						
Non‐Muslim	3.115	1.087–8.930	0.034**	1.040	0.256–4.224	0.957
Muslim^(R)^	1.00			1.00		
Caregivers' exposure to mass media						
No	0.483	0.252–0.925	0.028**	1.027	0.414–2.550	0.954
Yes^(R)^	1.00			1.00		
Education of the household head						
Non literate	2.916	0.768–11.078	0.116	0.537	0.069–4.167	0.552
Primary	1.329	0.480–3.678	0.584	0.484	0.116–2.022	0.320
Secondary	1.564	0.623–3.930	0.341	0.913	0.250–3.332	0.891
Higher education^(R)^	1.00			1.00		
Household assets						
Low	1.183	0.270–5.178	0.823	4.012	0.442–36.380	0.217
Moderate	1.148	0.293–4.502	0.843	1.950	0.234–16.272	0.537
High^(R)^	1.00			1.00		
**Interpersonal factors**						
Household food insecurity						
Food secure household	1.104	0.387–3.152	0.853	1.568	0.390–6.313	0.526
Mildly food insecure household	1.114	0.391–3.169	0.540	1.414	0.352–5.682	0.626
Moderately food insecure household	1.739	0.705–4.290	0.230	2.491	0.742–8.362	0.140
Severely food insecure household^(R)^						
Household vulnerability						
Low	0.192	0.054–0.676	0.010**	0.142	0.032–0.637	0.011**
Moderate	0.683	0.224–2.084	0.503	0.399	0.105–1.518	0.178
High^(R)^	1.00			1.00		
Household NGO membership						
No	2.968	1.536–5.734	0.001***	0.578	0.227–1.474	0.251
Yes^(R)^	1.00			1.00		
**Community‐level factors**						
Place of residence						
*Pankhali* village	2.333	0.161–33.789	0.535	10,737,809.608	0.000	0.990
*Hoglabunia* village	2.439	0.188–31.662	0.496	23,329,699.766	0.000	0.989
*Katabunia* village	2.301	0.173–30.585	0.528	28,254,711.077	0.000	0.989
*Sutarkhali* village	0.238	0.016–3.580	0.299	85,358,995.909	0.000	0.988
*Nolian* village	10.084	2.963–34.322	< 0.001***	18.381	3.721–90.803	< 0.001***
*Kalabogi* village^(R)^	1.00			1.00		
Frequency of natural disasters over the past 6 years						
≤ 6 events	1.369	0.659–2.843	0.400	0.918	0.301–2.800	0.880
≥ 7 events^(R)^	1.00			1.00		
**Policy‐level factors**						
Distance to upazila health complex						
< 10 km	0.605	0.052–7.039	0.688	3.085E‐007	0.000	0.990
10–20 km	0.556	0.058–5.342	0.611	1.388E‐007	0.000	0.990
> 20 km^(R)^	1.00			1.00		

Abbreviations: AOR = adjusted odds ratio; CI = confidence interval; R = reference category.

* Significant at 10% level.

** Significant at 5% level.

*** Significant at 1% level.

At the personal level, child age was found to be a significant determinant of wasting. Compared with children aged 49–59 months, those aged 6–12, 13–24, and 25–36 months had significantly lower odds of both moderate (AOR = 0.143; 95% CI: 0.047–0.433; *p* = 0.001; AOR = 0.176; 95% CI: 0.067–0.466; *p* < 0.001; AOR = 0.388; 95% CI: 0.160–0.939; *p* = 0.036) and severe wasting (AOR = 0.096; 95% CI: 0.020–0.465; *p* = 0.004; AOR = 0.198; 95% CI: 0.051–0.767; *p* = 0.019), respectively. Female children were more vulnerable, exhibiting 2.498 times higher odds of severe wasting than male children (AOR = 2.498; 95% CI: 1.134–5.504; *p* = 0.023). Furthermore, infant feeding practices were also influential. Children who were not exclusively breastfed during the early 6 months had significantly 0.064 times lower odds of severe wasting (AOR = 0.064; 95% CI: 0.012–0.343; *p* = 0.001). Additionally, children who were fed four times or less and 5–6 times daily had 0.121 times (AOR = 0.121; 95% CI: 0.029–0.505; *p* = 0.004) and 0.126 times (AOR = 0.126; 95% CI: 0.031–0.521; *p* = 0.004) lower odds of severe wasting compared to those fed ≥ 7 times daily.

At the interpersonal‐level, caregivers' education and occupation significantly influenced child wasting. Children of non‐literate caregivers faced substantially 6.695 times greater odds of moderate wasting (AOR = 6.695; 95% CI: 1.112–40.306; *p* = 0.038), while whose caregivers had primary education showed reduced odds of severe wasting (AOR = 0.258; 95% CI: 0.059–1.132; *p* = 0.073). Children of caregivers involved in farming, fishing, fish cultivation or bamboo crafting exhibited 14.333 times (AOR = 14.333; 95% CI: 0.798–257.551; *p* = 0.071) and 4.834 times (AOR = 4.834; 95% CI: 0.794–29.431; *p* = 0.087) greater odds of moderate wasting and those of housewives were at substantially higher risk of severe wasting (AOR = 4.352E‐012; 95% CI: 6.856E‐013–3.097E‐011; *p* < 0.001) compared to children of caregivers employed in business, jobs, or tailoring. In addition, caregivers with monthly income of BDT < 5000 had 0.178 times lower odds of moderate wasting (AOR = 0.178; 95% CI: 0.042–0.753; *p* = 0.019). Religion was also a significant determinant of wasting, with non‐Muslim children experiencing over threefold higher odds of moderate wasting than Muslim children (AOR = 3.115; 95% CI: 1.087–8.930; *p* = 0.034). Surprisingly, lack of exposure to mass media among caregivers was associated with 0.483 times lower odds of moderate wasting (AOR = 0.483; 95% CI: 0.252–0.925; *p* = 0.028).

At the household and community levels, lower household vulnerability was strongly protective, reducing the likelihood of both moderate (AOR = 0.192; 95% CI: 0.054–0.676; *p* = 0.010) and severe wasting (AOR = 0.142; 95% CI: 0.032–0.637; *p* = 0.011). Conversely, absence of household NGO membership increased the risk of moderate wasting nearly threefold (AOR = 2.968; 95% CI: 1.536–5.734; *p* = 0.001). Geographical disparities were also evident as children in *Nolian* village had 10.084 times and 18.381 times greater odds of both moderate (AOR = 10.084; 95% CI: 2.963–34.322; *p* < 0.001) and severe wasting (AOR = 18.381; 95% CI: 3.721–90.803; *p* < 0.001) compared to those residing in *Kalabogi* village.

### 5.4 Determinants of Underweight Among Children Under Five

Multinominal logistic regression analysis was conducted to identify the determinants of underweight among children under five (see Table 4) and categorized as normal weight (reference category), overweight, moderately underweight, and severely underweight. Explanatory variables included child, caregivers, household, community, and policy‐level factors. The findings showed that individual‐level factors such as child age, sex, and birth weight, along with interpersonal factors like caregivers' monthly income, were significantly associated with underweight. Furthermore, community‐level variables such as frequency of natural disasters over the past 6 years were found to be significant determinants of underweight prevalence. In contrast, policy‐level variables did not show any statistically significant association with underweight prevalence.

**TABLE 4:** Multivariate logistic regression analysis of underweight and its determinants.

Variables	Overweight			Moderately underweight			Severely underweight		
	AOR	95% CI (Lower‐upper)	*p*	AOR	95% CI (Lower‐upper)	*p*	AOR	95% CI (Lower‐upper)	*p*
**Individual factors**									
Age of the children									
6–12 months	17.144	0.258–1138.491	0.184	0.149	0.025–0.896	0.037**	0.255	0.037–1.749	0.164
13–24 months	0.977	0.017–54.662	0.991	0.464	0.077–2.809	0.403	0.847	0.126–5.718	0.865
25–36 months	3.776	0.093–154.096	0.595	0.258	0.052–1.265	0.095*	0.414	0.076–2.241	0.306
37–48 months	3.294	0.041–266.777	0.595	1.101	0.197–6.142	0.912	0.674	0.108–4.225	0.674
49–59 months^(R)^	1.00			1.00			1.00		
Sex of the children									
Girl	0.733	0.073–7.396	0.792	2.081	0.800–5.417	0.133	3.804	1.359–10.651	0.011**
Boy^(R)^	1.00			1.00			1.00		
Birth order									
1	0.313	0.005–19.434	0.581	0.572	0.105–3.111	0.518	0.788	0.131–4.734	0.794
2	1.120	0.034–36.447	0.949	1.089	0.197–6.014	0.922	1.145	0.188–6.968	0.883
≥ 3^(R)^	1.00			1.00			1.00		
Birth weight									
Underweight	1,013,099.679	0.000	0.992	1.343	0.031–58.031	0.878	51,993,840.563	6,696,541.946–403,694,844.040	< 0.001***
Normal weight	191,837.904	0.000	0.993	0.294	0.012–7.386	0.457	3,960,584.419	3,960,584.419–3,960,584.419	—
Overweight^(R)^	1.00			1.00			1.00		
Type of delivery									
Caesarian	3.748	0.433–32.468	0.230	1.736	0.630–4.784	0.286	1.223	0.416–3.628	0.716
Normal^(R)^	1.00			1.00			1.00		
Excusive breastfeeding in early 6 months									
No	0.278	0.008–9.473	0.477	0.873	0.234–3.256	0.840	0.491	0.110–2.204	0.353
Yes^(R)^	1.00			1.00			1.00		
Feeding frequency									
≤ 4 times	4.135	0.065–263.673	0.503	0.585	0.120–2.848	0.507	0.616	0.113–3.364	0.576
5–6 times	2.507	0.037–168.368	0.668	0.862	0.162–4.598	0.862	0.725	0.123–4.277	0.723
≥ 7 times^(R)^	1.00			1.00			1.00		
**Interpersonal factors**									
Caregivers' education									
Non literate	0.495	2.246E‐005–10,926.643	0.891	0.393	0.026–5.977	0.501	0.901	0.047–17.441	0.945
Primary	1.332	0.039–45.060	0.873	0.569	0.107–3.025	0.509	0.526	0.083–3.342	0.496
Secondary	0.437	0.015–13.104	0.633	0.698	0.155–3.139	0.640	0.763	0.143–4.070	0.752
Higher education^(R)^	1.00			1.00			1.00		
Caregivers' occupation									
Farming/fishing/fish cultivation	0.055	6.217E‐008–49,431.505	0.679	0.473	0.013–16.565	0.680	0.224	0.004–11.144	0.453
Day labor	0.040	8.796E‐010 –1,779,872.240	0.719	20.121	0.004–93373.556	0.486	15.152	0.003–79070.917	0.534
Housewife	0.075	2.716E‐009–2,044,785.480	0.766	6.550	0.041–1042.913	0.468	19.287	0.091–4067.049	0.278
Bamboo crafting	0.862	0.000–5351.367	0.973	0.920	0.044–19.275	0.957	0.426	0.017–10.651	0.603
Others (Business/job/tailoring)^(R)^	1.00			1.00			1.00		
Caregivers' monthly income (in BDT)									
No income	1.549	7.099E‐008 –33,788,801.017	0.960	0.546	0.004–69.705	0.807	0.070	0.000–11.905	0.311
< 5000	0.445	0.001–371.516	0.813	19.506	0.645–589.728	0.088*	5.738	0.163–201.749	0.336
≥ 5000^(R)^	1.00			1.00			1.00		
Religion									
Non‐Muslim	0.027	0.000–3.217	0.139	1.230	0.277–5.454	0.785	0.873	0.170–4.490	0.871
Muslim^(R)^	1.00			1.00			1.00		
Caregivers' exposure to mass media									
No	0.114	0.006–2.175	0.149	0.912	0.307–2.714	0.869	0.768	0.237–2.481	0.658
Yes^(R)^	1.00			1.00			1.00		
Education of the household head									
Non literate	7.257	0.003–18,663.288	0.621	9.587	0.424–217.024	0.156	2.876	0.118–70.126	0.517
Primary	2.436	0.025–232.881	0.702	3.326	0.701–15.774	0.130	1.309	0.243–7.048	0.754
Secondary	12.624	0.18–805.963	0.232	1.276	0.338–4.815	0.719	0.890	0.206–3.853	0.876
Higher education^(R)^	1.00			1.00			1.00		
**Interpersonal factors**									
Household assets									
Low	0.011	3.874E‐005–3.032	0.115	0.374	0.024–5.924	0.485	1.455	0.064–32.879	0.814
Moderate	0.007	4.572E‐005–1.101	0.055*	0.218	0.015–3.083	0.260	0.783	0.039–15.857	0.873
High^(R)^	1.00			1.00			1.00		
Household food insecurity									
Food secure household	0.797	0.015–43.454	0.911	0.893	0.174–4.590	0.892	0.771	0.130–4.583	0.774
Mildly food insecure household	1.583	0.043–57.677	0.802	0.985	0.169–5.735	0.987	2.324	0.363–14.881	0.374
Moderately food insecure household	1.529	0.054–42.964	0.803	0.809	0.172–3.803	0.789	1.383	0.266–7.189	0.700
Severely food insecure household^(R)^	1.00			1.00			1.00		
Household vulnerability									
Low	0.077	0.001–9.651	0.298	1.231	0.168–9.034	0.838	0.235	0.031–1.793	0.162
Moderate	0.986	0.031–30.913	0.994	1.963	0.292–13.170	0.488	0.406	0.05–2.787	0.359
High^(R)^	1.00			1.00			1.00		
Household NGO membership									
No	1.708	0.145–20.159	0.671	1.341	0.480–3.749	0.576	0.556	0.181–1.703	0.304
Yes^(R)^	1.00			1.00			1.00		
**Community‐level factors**									
Place of residence									
*Pankhali* village	0.775	2.123E‐007 –2,831,969.050	0.974	0.174	1.384E‐005–2188.659	0.717	0.026	1.897E‐006–350.209	0.451
*Hoglabunia* village	0.493	1.447E‐007–1,678,160.705	0.927	0.172	1.426E‐005–2082.691	0.714	0.025	1.943E‐006–331.756	0.447
*Katabunia* village	0.098	1.870E‐008 –511,057.248	0.768	0.110	8.763E‐006–1376.060	0.646	0.043	3.191E‐006–570.522	0.515
*Sutarkhali* village	0.944	1.886E‐007–4,726,635.757	0.994	0.014	1.162E‐006–157.607	0.368	0.004	3.567E007–54.923	0.260
*Nolian* village	0.613	0.008–45.980	0.824	2.818	0.442–17.973	0.237	1.510	0.217–10.498	0.677
*Kalabogi* village ^(R)^	1.00			1.00			1.00		
Frequency of natural disasters over the past 6 years									
≤ 6 events	0.521	0.015–18.203	0.719	3.418	0.831–14.065	0.089*	3.757	0.842–16.765	0.083*
≥ 7 events ^(R)^	1.00			1.00			1.00		
**Policy‐level factors**									
Distance to upazila health complex									
< 10 km	1.141	4.661E‐007–2,791,407.515	0.986	5.754	0.001–6302.401	0.713	22.313	0.002–264,244.846	0.516
10–20 km	0.951	6.723E‐007–1,344,692.941	0.994	30.487	0.004–264,859.560	0.460	66.259	0.007–595,945.593	0.367
> 20 km^(R)^	1.00			1.00			1.00		

Abbreviations: AOR = adjusted odds ratio; CI = confidence interval; R = reference category.

* Significant at 10% level.

** Significant at 5% level.

*** Significant at 1% level.

Children aged 6–12 and 25–36 months demonstrated 0.149 times (AOR = 0.149; 95% CI: 0.025–0.896; *p* = 0.037) and 0.258 times (AOR = 0.258; 95% CI: 0.052–1.265; *p* = 0.095) lower odds of moderate underweight compared to those aged 49–59 months. Female children were disproportionately affected, with nearly 3.8fold higher odds of severe underweight than male children (AOR = 3.804; 95% CI: 1.359–10.651; *p* = 0.011). Low birth weight was found as the strong predictor, with children born underweight facing dramatically elevated odds of severe underweight (AOR = 51,993,840.563; 95% CI: 6,696,541.946–403,694,844.040; *p* < 0.001) compared to those born overweight.

At the household level, children of caregivers with monthly income of BDT < 5000 had higher odds of moderate underweight than those with higher‐income caregivers (AOR = 19.506; 95% CI: 0.645–589.728; *p* = 0.088). Community‐level environmental stressors were also influential; children residing in the community experiencing six or fewer natural disasters within the past 6 years had greater odds of both moderate and severe underweight (AOR = 3.418; 95% CI: 0.831–14.065; *p* = 0.089; AOR = 3.757; 95% CI: 0.842–16.765; *p* = 0.083) than those in more frequently affected communities, suggesting differences in coping mechanisms or resource allocation.

## 6 Discussion

The study investigated the prevalence and determinants of undernutrition in the form of stunting, wasting, and underweight among children under five in the southwestern coastal region of Bangladesh. The findings revealed an alarmingly high burden of stunting, with 56.3% of children severely stunted and 33.3% moderately stunted. These results align with findings from previous studies in Bangladesh (57.5% severe and 29% moderate stunting) (Akter and Nishu 2025) and Nepal (56.7%) (Karki et al. 2023), yet substantially exceed the national stunting rate of 24% (National Institute of Population Research and Training (NIPORT) and ICF 2023) as well as the international prevalence rates from Pakistan (40%) (Soofi et al. 2023) and Ethiopia (38%) (Fenta, Workie, et al. 2020; Fenta, Tesfaw, and Derebe 2020). Similarly, the prevalence of wasting in the current study was notably high, with 40.5% of children moderately wasted and 16.4% severely wasted, far higher than the national rate of 11% (National Institute of Population Research and Training (NIPORT) and ICF 2023) and rates reported in Ethiopia (36.4%) (Ewune et al. 2022), India (17.1%) (Murarkar et al. 2020), and Nepal (10%) (Vijay and Patel 2024).

Moreover, we observed a high prevalence of underweight, with 59.2% moderately underweight and 28.7% severely underweight, compared to the national prevalence rate of 22% (National Institute of Population Research and Training (NIPORT) and ICF 2023) and lower levels than reported in neighboring countries like India (35.4%) (Murarkar et al. 2020), Pakistan (23.3%) (Siddiqa et al. 2023), and Nepal (27%) (Vijay and Patel 2024). These elevated rates in childhood undernutrition may be attributed to the distinct socioeconomic and environmental characteristics of the coastal region, including heightened vulnerability to climate‐related hazards, limited livelihood strategies, poor healthcare access, lower educational attainment, and cultural or suboptimal child feeding practices.

### 6.1 Determinants of Stunting, Wasting, and Underweight

Multidimensional factors were found to significantly influence the prevalence of stunting, wasting, and underweight among children under five in coastal Bangladesh. The regression analyses revealed that child age, birth weight, caregivers' occupation, mass media exposure, education of the household head, household food insecurity, vulnerability, and NGO membership were the significant determinants of stunting prevalence among children under five. Similarly, child age, sex, exclusive breastfeeding in the early 6 months, feeding frequency, caregivers' education, occupation, monthly income, and exposure to mass media, religion, household vulnerability, NGO membership, and place of residence were the significant determinants of wasting prevalence among children. Besides, age, sex, and birth weight of the children, caregivers' monthly income, and frequency of natural disaster over the past 6 years were the significant determinants of underweight prevalence among children under five. Guided by Urie Bronfenbrenner's Social Ecological Model (SEM), this study systematically classified the determinants of undernutrition into individual, interpersonal, community, and policy‐level factors.

#### 6.1.1 Individual Factors

Child age was identified as a significant determinant of stunting, wasting, and underweight prevalence among under‐five children in coastal Bangladesh. Children aged 6–12 months had lower odds of moderate stunting and underweight, while those aged 6–24 months and 25–36 months were less likely to experience moderate or severe wasting compared to children aged 49–59 months. These findings align with previous studies from Bangladesh, Nepal, and Ethiopia, which demonstrate an age‐related increase in stunting and underweight (Akter and Nishu 2025; Fenta, Tesfaw, and Derebe 2020; Khanam et al. 2019), although discrepancies exist regarding the age pattern of wasting (Abdulla et al. 2023; Hossain et al. 2022). The comparatively lower prevalence of stunting and underweight among younger cohorts likely reflects the protective effects of breastfeeding during the first two years in Bangladesh (Chowdhury et al. 2020). Conversely, the higher prevalence of stunting and underweight among older children may stem from inadequate nutrient intake and increased exposure to infectious diseases (Akter and Nishu 2025).

Gender‐based disparities were also observed, with female children showing higher odds of severe wasting and underweight than their male counterparts, corroborating earlier research that associates female disadvantage with entrenched gender inequality and male preference (Kumar et al. 2019; Manjong et al. 2021; Tarikujjaman 2023). However, these results diverge from studies reporting greater wasting among boys, often explained by biological and sociocultural dynamics (Abdulla et al. 2023; Thurstans et al. 2020; Wondimu and Dejene 2022). The observed variations may arise from the interplay of biological vulnerability, differential nutritional intake, and increased susceptibility to infections among female children (Sahiledengle et al. 2023). Furthermore, gendered sociocultural norms that privilege boys in feeding practices (Shah et al. 2020) and intra‐household food allocation, often providing males with larger portions or preferential access to nutrient‐dense foods (Coleman et al. 2019), may reinforce these disparities.

Birth weight was also identified as a critical determinant of childhood stunting and underweight. In line with prior studies (Chowdhury, Rahman, et al. 2022; Chowdhury, Chakrabarty, et al. 2022; Siddiqa et al. 2023; Tamir et al. 2024), the current study found that children with low birth weight faced significantly greater odds of moderate and severe stunting and underweight. The justification of this finding may be the influence of inadequate maternal nutrition during pregnancy, which leads to intrauterine growth restriction and subsequent long‐term growth deficits (Siddiqa et al. 2023). This finding highlights the critical need for targeted maternal nutrition and early childhood nutritional interventions.

Breastfeeding duration has been identified as a critical determinant of wasting among children under five. Interestingly, the present study revealed that children who were not exclusively breastfed during the first 6 months exhibited lower odds of severe wasting compared to their exclusively breastfed counterparts. This finding contrasts with prior evidence suggesting that exclusive breastfeeding reduces the risk of wasting in children under five (Ewune et al. 2022; Hossain et al. 2022). A plausible explanation of the findings of this study may be that non‐exclusive breastfeeding practices increase the likelihood of early introduction of formula milk, which could contribute to higher body weight in children and consequently lower susceptibility to wasting, a relationship that warrants further empirical exploration.

Consistent with existing research, this study found a significant relationship between complementary feeding practices and the prevalence of wasting among children (Saleem et al. 2024), with those fed ≤ 4 times or 5–6 times per day demonstrating lower odds of severe wasting compared to those fed ≥ 7 times daily. Ensuring adequate complementary feeding alongside continued breastfeeding during the first 2 years is essential for meeting nutritional requirements, promoting healthy growth, and mitigating the risk of malnutrition (Kahssay et al. 2020; World Health Organization 2021). Nevertheless, these findings contrast with previous studies indicating a higher likelihood of wasting among children receiving fewer daily meals (Chekol et al. 2022; Yigezu et al. 2024). This discrepancy highlights that not merely feeding frequency, but also the quality, nutrient density, and timing of complementary foods are crucial for child growth, development, and overall survival.

#### 6.1.2 Interpersonal Factors

Parental education was found as a significant determinant of wasting prevalence among children under five, with children of non‐literate caregivers exhibiting greater odds of moderate wasting compared to children of educated parents, consistent with prior research (Aheto 2020; Hossain et al. 2022; Sharaf et al. 2019). This association may be explained by the fact that education enhances caregivers' knowledge of nutrition and health, facilitates income‐generating opportunities, and promotes positive childcare practices, including appropriate feeding, vaccination, and hygiene management (Islam et al. 2020; Sharaf et al. 2019). Additionally, higher maternal education supports empowerment and decision‐making autonomy (Akter, Chanda, et al. 2018), contributing to reduced malnutrition prevalence among children in Bangladesh.

Caregivers' occupational status also significantly influenced childhood stunting and wasting. Children of housewives demonstrated greater odds of severe stunting and wasting than those whose caregivers were engaged in business, job, or tailoring. This corresponds with prior studies linking non‐working mothers to increased stunting (Akter and Nishu 2025) and wasting risks (Musa et al. 2024; Siddiqa et al. 2023). A potential interpretation for this may be that greater autonomy, health literacy, and healthcare access among employed and educated women (Akter, Jabbar, and Khatun 2018; Sarma et al. 2017) enhance childcare practices. Conversely, unemployment among rural women, often tied to limited career aspirations (Akter, Jabbar, and Khatun 2018) and financial constraints, can reduce their capacity to ensure adequate nutrition and healthcare for children, thereby exacerbating malnutrition risks (Akter 2022).

Household head's education was identified as protective against child undernutrition. Children in households where the head had only primary education showed higher odds of moderate stunting compared with those from households led by heads with higher education, corroborating prior findings (Chowdhury, Rahman, et al. 2022; Chowdhury, Chakrabarty, et al. 2022; Sultana et al. 2019). This can be attributed to the fact that educated parents are likely to possess greater knowledge and awareness of nutrition, hygiene, and health services, in addition to improved income levels, which facilitate access to diverse and nutritious foods (Sultana et al. 2019).

This study found parental income as a key determinant of child nutritional outcomes, with children from households earning less than BDT 5000 per month exhibiting higher odds of moderate underweight than those from higher‐income households, consistent with previous studies (Chowdhury et al. 2018; Hasan et al. 2020). Interestingly, children from low‐income households showed lower odds of moderate wasting compared with higher‐income peers, contrasting prior findings linking low income to increased wasting prevalence (Khanam et al. 2019; Kumar et al. 2021; Saleem et al. 2024). Limited household income restricts access to diverse, nutrient‐rich foods and healthcare services, highlighting broader socioeconomic inequalities that perpetuate childhood undernutrition in resource‐constrained coastal settings.

Religion was also evident as a significant determinant of child undernutrition and the current study found that non‐Muslim children experienced higher odds of moderate wasting than Muslim children, consistent with studies from India (Banerjee and Shirisha 2023; Kundu et al. 2024). This disparity reflects differences in dietary patterns, cultural practices, and healthcare access among various religious communities. This may be justified that religious and cultural food taboos, particularly those restricting animal‐source foods during pregnancy, lactation, and early childhood, contribute to maternal undernutrition and subsequent child stunting (Gebregziabher et al. 2023; Kumar et al. 2018). This result highlights the importance of culturally sensitive nutritional interventions.

Exposure to mass media was inversely associated with stunting, with children of caregivers without media exposure demonstrating higher odds of moderate stunting, corroborating prior findings (Huo et al. 2022; Jung et al. 2025). The explanation of this finding may be that mass media likely enhances caregivers' awareness of hygiene, appropriate feeding, and health practices critical for child growth. However, unexpectedly, the present study found that children of caregivers without media exposure exhibited lower odds of moderate wasting, diverging from previous studies (Oswal et al. 2025; Tamanna et al. 2025). This inconsistency may reflect that excessive media engagement reduces time and attention for direct childcare activities, such as meal preparation and feeding frequency, thereby influencing nutritional outcomes warranting further investigation.

Moreover, the present study documented household food security as a protective factor, with children from food secure households showing lower odds of severe stunting, consistent with existing research (Na et al. 2020; Patriota É et al. 2024). This might be explained as that household food security guarantees adequate access to sufficient, diverse, and nutrient‐rich foods during early childhood, mitigates the risk of chronic malnutrition and supports optimal growth.

Household vulnerability was another significant determinant of stunting and wasting prevalence, with children from less vulnerable households exhibiting lower odds of moderate and severe wasting, and those from moderately vulnerable households showing lower odds of severe stunting relative to children from highly vulnerable households. This aligns with existing literature indicating that children from poorer households face elevated stunting and wasting risks compared to children from affluent households (Kundu et al. 2024; Li et al. 2020; Utumatwishima et al. 2024; Wali et al. 2021). This can be interpreted as that households' vulnerability reflects socioeconomic status, where typically, poor households are more vulnerable than wealthier households. Therefore, households with limited income often underinvest in nutrition, making them more vulnerable to growth failure due to inadequate food and substandard living conditions (Wali et al. 2021).

Moreover, NGO membership was identified as a crucial predictor of childhood stunting and wasting. Children from non‐member households had greater odds of stunting and wasting, consistent with prior evidence from Bangladesh (Khanam et al. 2019). The justification of this finding is that NGO participation provides households with access to financial support to address different challenges, including purchasing nutritious food, providing better healthcare for children, and maintaining a healthier living environment. Moreover, NGO participation enhances women's empowerment and decision‐making, ultimately improving child nutritional outcomes (Heckert et al. 2019; Poudel et al. 2022).

#### 6.1.3 Community‐Level Factors

In alignment with earlier studies (Abdulla et al. 2023; Khanam et al. 2019; Wondimu and Dejene 2022), the present study found that place of residence exerts a significant influence on childhood wasting. Children residing in *Nolian* village exhibited greater odds of both moderate and severe wasting compared with those in *Kalabogi* village. These spatial disparities in children's nutritional outcomes can be explained by the underlying variations in socioeconomic conditions, cultural practices, social support, and access to maternal and child healthcare services in the coastal communities. Moreover, geographic and infrastructural factors such as road and transportation systems, market accessibility, food availability and pricing, dietary diversity, land scarcity, and healthcare infrastructure further contribute to intra‐regional differences supported by existing literature (Rahman and Hossain 2022; Sharaf et al. 2019; Wondimu and Dejene 2022).

Furthermore, children living in communities frequently affected by natural disasters exhibited significantly higher odds of underweight, aligning with previous research highlighting that recurrent environmental shocks and natural disasters such as floods, cyclones, and salinity intrusion intensify child malnutrition (Hussain and Sharma 2025; Petscavage et al. 2025). This finding may be attributed to environmental stressors in coastal Bangladesh, which undermine agricultural productivity, deplete fisheries resources, and reduce dietary diversity, thereby exacerbating food insecurity and nutritional deficiencies. Global research also confirms that climate variability and escalating food prices disproportionately affecting poor households, increasing the risk of childhood stunting and wasting (Fanzo et al. 2018; Lloyd et al. 2018; Yadav et al. 2024). Consequently, the persistent exposure to climatic hazards not only compromises immediate nutritional outcomes but also entrenches long‐term vulnerability to undernutrition among children in disaster‐prone coastal regions.

#### 6.1.4 Policy‐Level Factors

Healthcare accessibility is a critical determinant of child undernutrition. Evidence from rural Pakistan indicates a significant association between distance to health facilities and child undernutrition, reporting higher odds of stunting and underweight among households situated ≥ 3 km from the nearest health facility compared to those living closer, highlighting the role of geographic barriers in malnutrition (Shahid et al. 2022). This finding suggests that greater distance may impede timely access to healthcare services, thereby elevating the risk of malnutrition. In contrast, the present study in coastal Bangladesh observed no significant relationship between distance to health facility and child undernutrition, consistent with a study from Uganda (Lubbers et al. 2025). This discrepancy may reflect contextual differences, including variations in rural and peri‐urban settings, transportation infrastructure, and the quality and readiness of health services, which can moderate the impact of distance on nutritional outcomes. Therefore, these observations emphasize that physical proximity alone may not fully capture healthcare accessibility or its influence on child nutritional outcomes, highlighting the need for further context‐specific investigations.

## 7 Strengths and Limitations

The key strength of this research is its community‐based approach which enables a comprehensive assessment of the three globally accepted indicators of child undernutrition such as stunting, wasting, and underweight among children under five in the southwestern coastal region of Bangladesh. Notably, this study employs the Social Ecological Model as a theoretical framework to systematically explore determinants of child undernutrition across individual, interpersonal, community, and policy levels. The use of random sampling further enhances the scientific rigor of the study design and minimizes the risk of selection bias, thereby strengthening the reliability and generalizability of the findings. Additionally, this study offers integrated context‐specific and multi‐level recommendations for addressing child undernutrition at local, national, and global levels. However, this study has a number of shortcomings. Firstly, this is a cross‐sectional study which restricts the causal relationship. Secondly, reliance on caregiver‐reported information for variables such as birth weight and child health history introduces potential recall bias.

## 8 Conclusion

The study aimed to investigate the prevalence and determinants of stunting, wasting, and underweight among children under five in the coastal region of Bangladesh. Findings revealed a high burden of stunting, wasting, and underweight among children, driven by a combination of personal, interpersonal, and community‐level factors. Child age, sex, birth weight, exclusive breastfeeding practices, feeding frequency, caregivers' education, occupation, income, mass media exposure, religion, education of the household head, household food insecurity, vulnerability, NGO membership, residence, and natural disaster exposure were the significant determinants of undernutrition among children under five. Based on the findings, this study emphasizes the urgency of implementing integrated, multi‐level strategies to combat child undernutrition at local, national, and global levels. Locally, community‐based nutrition programs should prioritize children aged 6–36 months and low‐birth weight infants while promoting exclusive breastfeeding, optimal complementary feeding, maternal education, and livelihood opportunities for women in disaster‐prone coastal areas. At the national level, embedding nutrition services into maternal and child health policies and programs, alongside poverty reduction, food security, social protection, and gender‐responsive nutrition policies, is essential. Globally, the study highlights the importance of climate‐resilient and context‐specific nutrition policies that address structural vulnerabilities. International organizations such as WHO, UNICEF, and WFP should support these efforts by integrating evidence into global nutrition frameworks and fostering cross‐country collaborations to advance equitable and sustainable child health outcomes.

## Author Contributions

All authors contributed substantially to this research and approved the final manuscript. S.A. conceptualized the study, supervised collection, management, analysis of data, and drafted the initial manuscript. A.S. served as the advisor and provided overall supervision, while A.A. and W.B. contributed as co‐advisors and supported study oversight. A.S., A.A., and W.B. critically evaluated and revised the manuscript. S.A., as the corresponding author, had full access to all study data and assumed overall responsibility for the final submission.

## Funding

The authors have nothing to report.

## Ethics Statement

The Committee of Research Ethics at the Faculty of Public Health, Chiang Mai University in Thailand granted ethics approval for the study, with the reference number ET020/2024. Furthermore, all participants provided written informed consent to take part in this research. The participants were guaranteed that their data would be treated with confidentiality, remain anonymous, and solely be used for research purposes.

## Consent

All authors gave their approval for the final version for submission.

## Conflicts of Interest

The authors declare no conflicts of interest.

## Data Availability

The data that support the findings of this study are available from the corresponding author upon reasonable request.
